# P-371. Mycobacterium Scrofulaceum Pseudo-Outbreak due to Laboratory Cross-Contamination

**DOI:** 10.1093/ofid/ofae631.572

**Published:** 2025-01-29

**Authors:** Patricia D Zuccaro, Emily landon, R Marrs, Vera Tesic, Vera Chu, Kathleen G Beavis, Nedra Love, Aurea Enriquez, Boghos Taslakjian, Allison H Bartlett

**Affiliations:** University of Chicago, Chicago, Illinois; University of Chicago, Chicago, Illinois; University of Chicago Medicine, Chicago, Illinois; University of Chicago, Department of Pathology, Chicago, Illinois; UChicago Medicine, Chicago, Illinois; University of Chicago, Chicago, Illinois; University of Chicago, Chicago, Illinois; University of Chicago, Chicago, Illinois; University of Chicago, Chicago, Illinois; University of Chicago Comer Children's Hospital, Chicago, IL

## Abstract

**Background:**

Mycobacterium scrofulaceum is a non-tuberculous mycobacteria (NTM) that has been recovered from numerous sources. The species rarely causes adult disease in immunocompetent hosts, but has been closely linked to pediatric cervical lymphadenitis as well as disease in the lungs, GI tract, and soft tissues of immunocompromised hosts. We report an M. scrofulaceum pseudo-outbreak due to sample cross contamination, which has not been previously reported.

**Methods:**

University of Chicago Medical Center is a tertiary care center containing an on-site microbiology lab. In March, 2023 the Infectious Diseases consultation service reported multiple M. scrofulaceum cases to the infection prevention and control program which prompted an investigation. Twenty-five positive cultures from seven different patients were reviewed.

Given the rarity of M. scrofulaceum infection, cross-transmission was suspected. Chart review was conducted by a multi-disciplinary team in order to determine if true infection occurred and identify potential epidemiological overlap.

**Results:**

Positive samples originated from soft tissue, lymph nodes, bone, sputum, and pleural fluid. Six patients improved clinically without treatment for M. scofulaceum.

The final patient had underlying hidradenitis suppurativa, pyoderma gangrenosum, and dermatosis artefacta requiring frequent hospitalization and debridement. M. scrofulaceum was isolated from deep tissue. Given the severity of the patient’s disease, frequent healthcare exposure, and extensive surgical history, he was highly suspected to be the source patient of the pseudo-outbreak. Upon review, we confirmed that most of the other positive samples were processed that same day.

**Conclusion:**

We present a series of 25 positive culture results growing mycobacterium scrofulaceum isolated from seven distinct patients. An investigation into potential cross-contamination within our mycobacteriology laboratory was conducted and confirmed our suspicion. This led to internal review of our standard processes and re-training of our laboratory personnel.

**Disclosures:**

**Allison H. Bartlett, MD, MS**, CVS/Caremark: Advisor/Consultant

